# Exploring the mystery of colon cancer from the perspective of molecular subtypes and treatment

**DOI:** 10.1038/s41598-024-60495-8

**Published:** 2024-05-13

**Authors:** Wenhong Lu, Qiwei Wang, Lifang Liu, Wenpeng Luo

**Affiliations:** 1 The Second Affiliated Hospital of Hunan University of Chinese Medicine, Changsha, 410005 Hunan People’s Republic of China; 2 Hunan Provincial Rehabilitation Hospital, Changsha, 410007 Hunan People’s Republic of China; 3 The First Affiliated Hospital of Hunan University of Chinese Medicine, Changsha, 410007 Hunan People’s Republic of China

**Keywords:** Non-negative matrix factorization, Unsupervised cluster, Colon cancer, Molecular subtypes, Cell cycle, Biotechnology, Cell biology, Immunology, Gastroenterology, Molecular medicine, Oncology

## Abstract

The molecular categorization of colon cancer patients remains elusive. Gene set enrichment analysis (GSEA), which investigates the dysregulated genes among tumor and normal samples, has revealed the pivotal role of epithelial-to-mesenchymal transition (EMT) in colon cancer pathogenesis. In this study, we employed multi-clustering method for grouping data, resulting in the identification of two clusters characterized by varying prognostic outcomes. These two subgroups not only displayed disparities in overall survival (OS) but also manifested variations in clinical variables, genetic mutation, and gene expression profiles. Using the nearest template prediction (NTP) method, we were able to replicate the molecular classification effectively within the original dataset and validated it across multiple independent datasets, underscoring its robust repeatability. Furthermore, we constructed two prognostic signatures tailored to each of these subgroups. Our molecular classification, centered on EMT, hold promise in offering fresh insights into the therapy strategies and prognosis assessment for colon cancer.

## Introduction

Colon cancer ranks among the most prevalent malignancies worldwide. Remarkable strides in its diagnosis and treatment have led to a marked reduction in mortality in recent years. However, the underlying mechanisms driving colon cancer remains elusive. In the current landscape of precision medicine, the categorization of molecular subtypes, characterized by distinct molecular attributes, plays a pivotal role in guiding treatment decisions and shaping prognostic outcomes for patients. For instance, breast cancer patients are routinely categorized into subtypes defined by estrogen and progesterone receptor expression levels. These subtypes confer varied risk profiles, dictating tailored therapeutic strategies. This highlights the significant clinical utility of molecular classification in breast cancer^1^. The pathogenesis of CRC is still unclear, and the classification of CRC is still mainly based on TNM staging, which is insufficient to understand CRC. Therefore, clarifying the molecular mechanisms of colon cancer occurrence can help the diagnosis and treatment of CRC. In recent years, machine learning (ML)-based approaches to understanding tumors have attracted more and more attention^2–4^, and many algorithms for predicting and classifying tumors have emerged^5–7^. Existing machine learning algorithms include linear regression, logistic regression, decision trees, support vector machines (SVM), naive Bayes, K-means clustering, random forests, dimensionality reduction algorithms, gradient boosting, and AdaBoost. Jiang et al. used convolutional neural networks to predict the prognosis of stage III CRC^8^. In the current context of precision medicine, the classification of molecular subtypes characterized by unique molecular properties plays a key role in guiding treatment decisions and shaping patient prognosis. Molecular subtypes have been used to explore colon heterogeneity. The gene expression subtypes of COAD and COADREAD were proposed by the Cancer Genome Atlas (TCGA) research network respectively^9,10^. A recent study also identified molecular subtypes of colon cancer based on multiple platforms, including molecular subtypes of COAD patients^11^, and several molecular subtypes of colon cancer based on different gene sets^12^. However, early colon cancer has some special molecular characteristics, and in these subtypes and gene sets, the prognostic information of patients is not well used, resulting in weak prognostic ability of patients. The consensus molecular subtypes (CMS) criteria for classifying colon cancer has garnered relative recognition^1^ but it does not yield a similar clinical impact as seen in breast cancer. This discrepancy implies potential limitations in the clinical applicability of CMS may, underscoring the urgent necessity to discover alternative molecular subtypes that can inform treatment decisions and prognostic assessments for colon cancer.

During tumor development, epithelial cells undergo a transformation known as the epithelial-to-mesenchymal transition (EMT), adopting mesenchymal characteristics such as enhanced motility, invasiveness, and resistance to apoptosis^13^. Epithelial–mesenchymal transition (EMT) is a complex cellular process in which epithelial cells acquire a mesenchymal phenotype. According to the physiological and histological context, EMT is divided into three types^14^: embryonic development and organ formation^15,16^ wound healing and organ fibrosis; and cancer progression^17–19^. Type 3 EMT is associated with an invasive or metastatic phenotype^20^. In the past decade, an increasing number of studies have provided strong evidence that EMT plays a crucial role in the progression and metastasis of various malignant tumors, including CRC^21^. During EMT, tumor cells undergo tight junction dissolution, apical-basal polarization destruction, and cytoskeletal structure reorganization, enabling the cells to develop an invasive phenotype. In cancer cells, EMT is abnormally regulated by extracellular stimuli from the tumor microenvironment, including growth factors and inflammatory cytokines, as well as physical stress within the tumor, such as hypoxia^22^. Therefore, EMT programming can enable tumor cells to adapt to the constantly changing human tumor microenvironment for successful metastasis. Preventing or reversing the lethal effects of EMT is crucial for cancer therapy. At present, there are three main strategies for the treatment of EMT^23^. First, it can inhibit tumorigenesis by blocking upstream signaling pathways. This includes ligand-neutralizing antibodies, decoy receptors or inhibitors that block TGFβ, NF-κB, EGFR, cMET, WNT and Notch signals^12–34^. In addition, effective inducers of EMT include a variety of proinflammatory signals, such as TNF-α^35^. Another therapeutic strategy is targeting the molecular drivers of EMT. Although EMT-TFs are the main drivers/regulators of the EMT process, direct targeting of transcription factors (EMT-TFs) is challenging^36^. Moreover, several EMT-TFs have complementary and redundant functions due to their tightly connected through feedback mechanisms. Therefore, targeting their interactions with important cofactors while targeting multiple EMT-TFs may be a more beneficial strategy^36^. The transformation of EMT involves the reprogramming of gene expression, primarily driven by signaling pathways responsive to extracellular cues. Among these pathways, transforming growth factor (TGF)-β signaling holds a predominant role, although the convergence of multiple signaling pathways is essential for inducing EMT^24^. Previous studies have implicated EMT-related signaling pathways in colon cancer, including Wnt^25^, TGF-β^26^, Hedgehog^27^, and Notch pathways^28^. Interestingly, EMT status has been linked to peritoneal metastasis, progression-free survival (PFS) and overall survival (OS) in ovarian cancer^29^. Furthermore, recent research has underscored the prognostic potential of EMT-related genes in neuroblastoma, ependymomas^30^, and bladder cancer^31^. However, a comprehensive analysis of their prognostic ability in colon cancer is currently lacking.

In this study, we identified two distinct molecular subtypes using genes related to EMT. These subgroups exhibited disparities in clinical features, OS, and genetic mutations. By employing nearest template prediction (NTP), we demonstrated the robust repeatability of our molecular classification within the original dataset and validated it across different datasets. This underscores the reliability of our classification approach, which centers on EMT, and offers promising new insights into colon cancer treatment and prognosis.

## Materials and methods

### Datasets and samples

We collected data from four Gene Expression Omnibus (GEO) datasets, specifically GSE17536 (55 stage II and 56 stage III patients with a median RFS of 3.0625 years; the 1-, 2-, and 3-year recurrence rate were 6.3%, 15.3%, and 23.4%, respectively), GSE29623 (Primary fresh frozen tissues from 65 patients (40 male and 25 female) with a mean age 65 ± 13 years and with AJCC Stages I (n = 7), II (n = 22), III (n = 18) and IV (n = 18) colon cancers, underwent RNA extraction and miRNA array analysis). and GSE71187 (189 samples with detail clinical information), all of which contained detailed survival information. Clinical information was obtained from the Cancer Genome Atlas Colon Adenocarcinoma (TCGA-COAD) project via the TCGA database. Potential batch effects were mitigated using the “Combat” package. GSE29621 and GSE39582 datasets were used to validate model accuracy.

### Gene set variation analysis (GSVA)

The Hallmarker pathway is applied to help researchers gain a deeper understanding of gene function and regulatory relationships in the genome. Through the GSEA Hallmarker pathway, researchers can discover which genes are co-regulated in specific biological processes, revealing the function and mechanism of action of these genes in organisms. There are great significance for the study of biological processes, disease mechanisms, and the design of drug treatment regimens. For pathway analyses, we leveraged the 50 hallmark pathways from the molecular signature database, sourced from the “GSEABase” package. To streamline our analysis and minimize pathway overlap and redundancy among pathways, the gene sets associated with these pathways were trimmed, ensuring the uniqueness of genes within each pathway. GSVA gene set variant analysis is an analytical method that enriches the gene set of microarray and RNA-seq data under parametric and unsupervised conditions. GSVA converts a gene-sample data matrix (microarray data, FPKM, RPKM, etc.) into a gene set-sample matrix. Based on this matrix, the enrichment of gene sets (such as KEGG pathway) in each sample can be further analyzed. Since the results of GSVA are a gene set-sample enrichment matrix, there is more freedom in downstream analysis than other gene set enrichment methods such as GSEA (Gene Set Enrichment Analysis). Subsequently, the GSVA package^23^ (version 1.22.4) was employed to estimate pathway scores (Table S6).

### Characterizing molecular subtypes and prognosis with immune cell data analysis

Two datasets encompassing gene expression matrices and data on the infiltration of 22 immune cells were used to confirm the existence of molecular subtypes. Gap statistics^15^ were utilized to determine the optimal number of subtypes and clustering prediction indices^32^ were used to assess the quality of cluster separation. Leveraging the capabilities of the “MOVICS” package in conjunction with the two datasets, we identified two distinct molecular subtypes with varying prognostic implications.

### Immunohistochemistry

Formalin-fixed, paraffin-embedded tissue sections were routinely dewaxed and hydrated, and then antigen was extracted at 120 °C for 3 min in 10 mM citric acid buffer (pH 6.0). The sections were then treated with 3% hydrogen peroxide at room temperature for 15 min, washed with phosphate buffer saline (PBS), and blocked with 10% goat serum for nonspecific binding. The sections were incubated overnight at 4 °C with a 1:2000 dilution of primary BGN antibody (Proteintech: Cat No:67275-1-Ig; china), then incubated with biotin-labeled secondary antibody at room temperature for 15 min, stained with 3,3-diaminobenzidine (DAB), and red-stained with hematoxylin.

### Differentially expressed genes (DEGs) linked to molecular subtypes

The “limma” package was employed for the identification of DEGs between the two subtypes. We considered statistical significance to be present when *P* < 0.01 and the absolute fold-change > 1.

### Module identification and analysis

The weighted gene co-expression network analysis (WGCNA) R package was employed to identify modules and study the relationships between them and the two clusters^33^. The optimal soft threshold was determined adhering to the scale-free topology criterion. Once it was determined, we defined the minimum module size as 30 genes. The identification of modules was accomplished using the dynamic tree cut method, with the MEDissThres parameter set at 0.25.

### Gene correlation and signature scoring for subtype characterization

Pearson correlation analysis was conducted for exploring the correlation between clusters and gene expression, as well as the activity of 50 hallmark pathways. Genes showing a positive correlation with the clusters (correlation coefficient > 0.4) were designated as “signature A”, while those with a negative correlation (correlation coefficient <  − 0.4) were classified as “signature B”. Subsequently, we applied the single-sample gene-set enrichment analysis (ssGSEA) algorithm to the 78 genes in signature B to construct a score that quantifies the impact of these genes on the two subtypes.

### Statistical analysis

Data analyses were performed using R software (v. 4.10). Differences between two subtypes were explored using the Wilcoxon test. The degree to which two subtypes were measured using the Pearson correlation coefficient. Threshold for statistical significance was *P* < 0.05.

### Ethics approval and consent to participate

All patients included in the study signed an informed consent form, and the study was approved by the Medical Ethics Committee of The Second Affiliated Hospital of Hunan University of Chinese Medicine (Ethics No : 2023-70); The study was carried out in accordance with the recommendations contained in the Declaration of Helsinki.

## Results

### Two molecular subtypes

**Figure 1:**
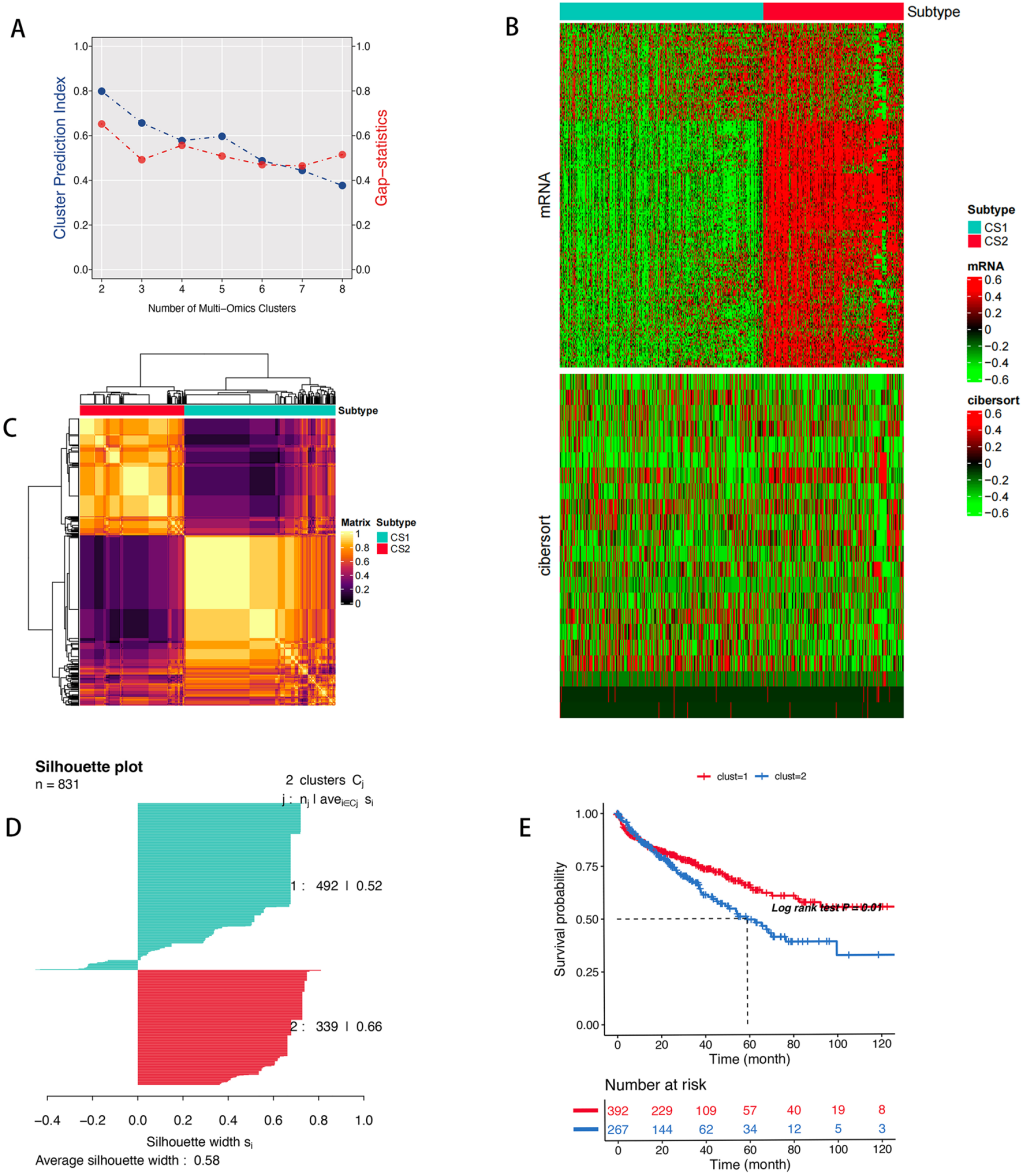
(**A**) The identified of clustering number by the CPI analysis and gap-statistical. (**B**) Consensus matrix based on the various algorithms. (**C**) The landscape of various data of 200 genes expression, 23 immune cells in different molecular subtypes. (**D**) Silhouette-analysis evaluation. (**E**) Survival analysis of the two clusters.

Clustering analysis was conducted using the “MOVICS” package, which incorporates various methods such as iClusterBayes, moCluster, CIMLR, IntNMF, ConsensusClustering, COCA, NEMO, PINSPlus, SNF, and LRA. These methods were applied to two datasets, which include the expression matrix of EMT-related genes expression matrix and data on the infiltration of 22 immune cells. The outcomes of CPI and Gaps analyses led us to identify two distinct molecular subtypes characterized by varying prognostic outcomes (Fig. 1A,C) and comparable scores (Fig. 1D). The cluster B showed better survival rate (Fig. 1E) than the cluster A. The distribution of multi-omics data among these subtypes is presented in Fig. 1B.

### Co-expression module identification

**Figure 2:**
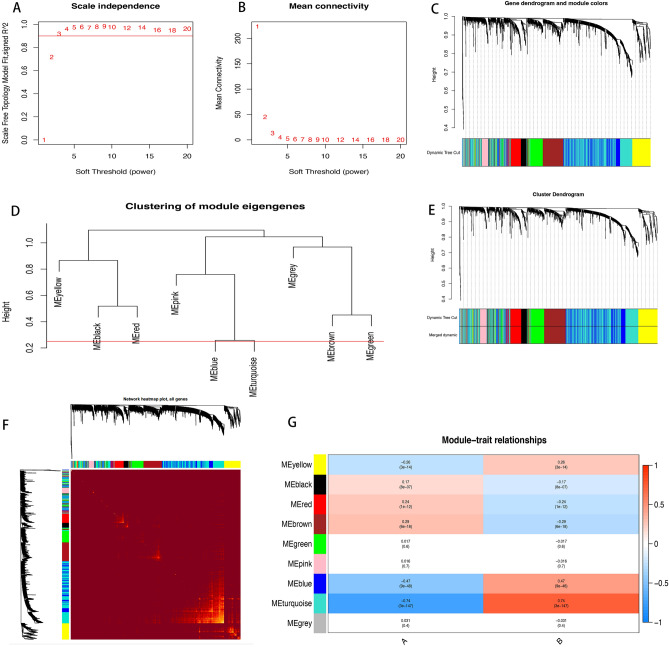
(**A**) Confirming the best scale-free index for various soft-threshold powers (β). (**B**) The mean connectivity for various soft-threshold powers. (**C**) The gene tree map and nodule color. (**D**) Hierarchical clustering analysis. (**E**) The gene dendrogram is based on clustering. (**F**) The heatmap of all genes. (**G**) Heatmap of the correlation between the module genes and the two clusters.

With a selected soft threshold power of β = 12 (Fig. 2A,B), we constructed phylogenetic trees to uncover co-expression modules (cut height ≥ 0.25) (Fig. 2C). Hierarchical clustering analysis of each module revealed that modules within the same branch exhibited similar patterns of gene expression (Fig. 2D). Consequently, similar gene modules were merged, resulting in the identification of 9 co-expression modules labeled by colors (yellow, black, red, brown, green, pink, blue, and turquoise) (Fig. 2E). Furthermore, we visualized gene clustering and assessed correlations among these modules (Fig. 2F). The magenta module exhibited a strong correlation with cluster B, warranting its selection as a crucial module for further analysis (Fig. 2G, Table S2).

### Signatures of molecular subtypes

**Figure 3:**
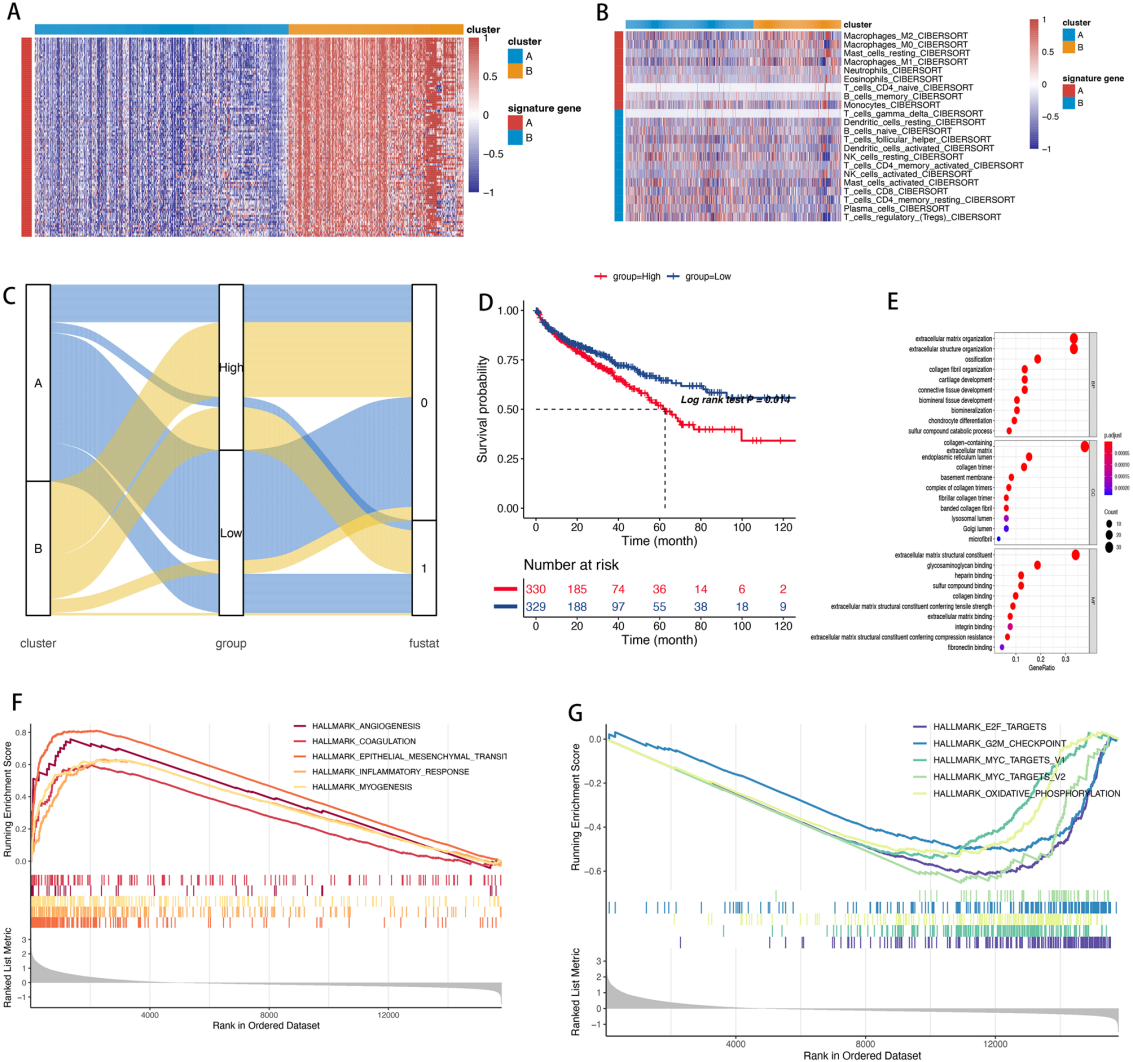
(**A**) Clustering analysis in DEGs of two clusters. (**B**) Clustering analysis in 22 immune cells of two clusters. (**C**) The distribution of clusters, and survival outcome in different score groups. (**D**) Survival analysis of the two score groups. (**E**) The biological function of signature A genes. (**F,G**) GSEA analysis based on 50 shallmark gene sets in two score groups.

Through Pearson correlation analysis, we identified correlations with a coefficient greater than 0.4 as “signature A” (Fig. 3A), and those with a coefficient less than − 0.4 as “signature B” (Fig. 3B). The signature A genes were enriched in the positive regulation of cell proliferation (Fig. 3E). Building upon the genes in signature A, the ssGSEA algorithm was utilized to quantify the clusters. Figure 3C illustrated a pronounced association of the high-score group with cluster B. Intriguingly, the low-score group displayed a more favorable survival rate compared to the high-score group (Fig. 3D). Our results underscore the effectiveness of this scoring model in evaluating the worst prognostic outcomes, primarily caused by the heightened activity of pathways related to tumor progression, including EMT, angiogenesis, coagulation, and myogenesis (Fig. 3E–G).

### Genetic and epigenetic event between different score groups

**Figure 4:**
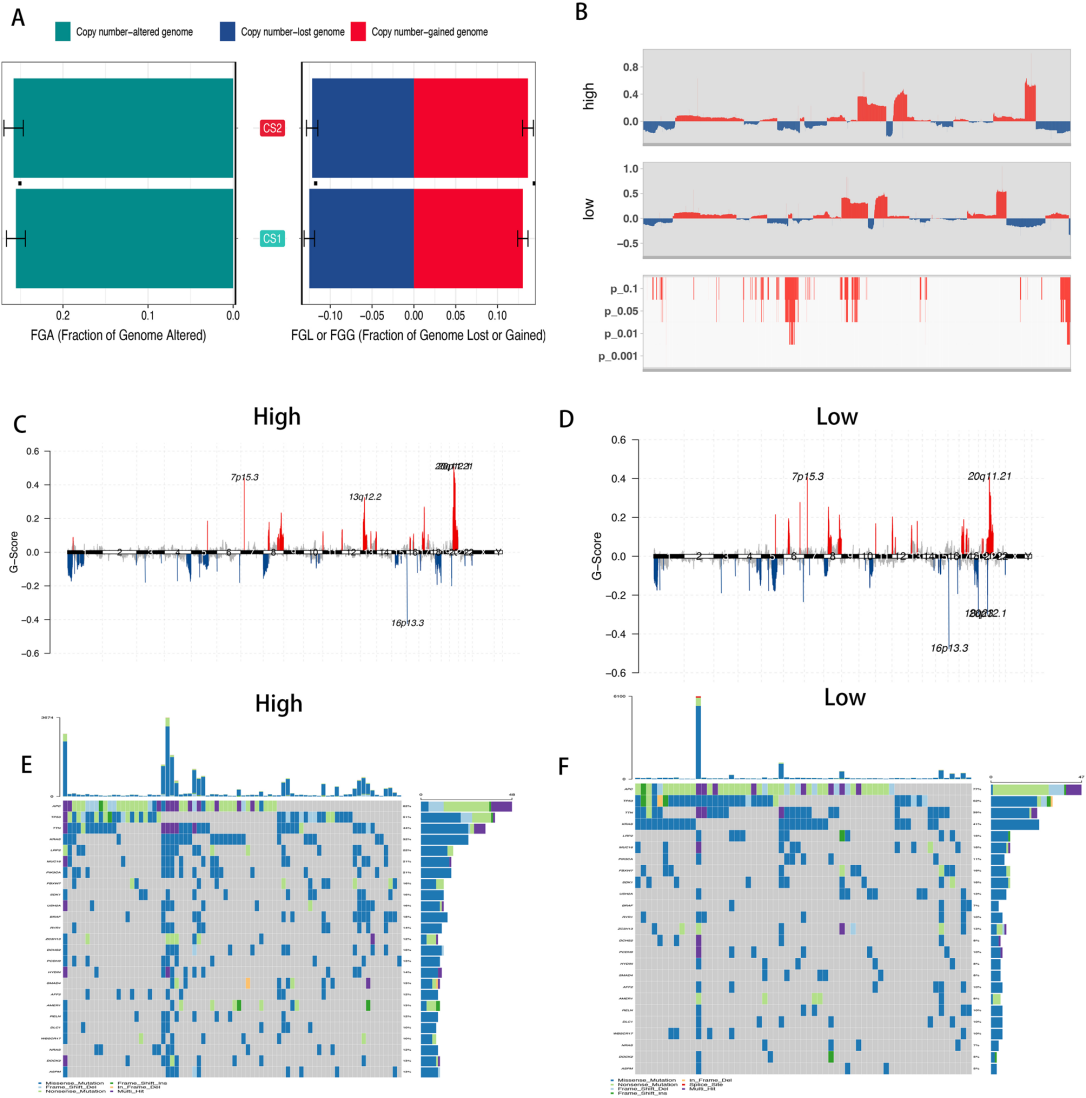
(**A**) The distribution of fraction genome altered (FGA) and fraction genome gain/loss (FGA/FGG) in the different score group. (**B**) CNA plot showed the relative frequency of copy number gains (red) or deletions (blue) between the high score group and low score group of the COAD cohort. (**C,D**) The distribution of the copy number of genomic regions between the high score group (**C**) and the low score group (**D**). (**E,F**) The significant mutation genes in the high scores (**D**) and low scores (**E**).

A newly study shown that the genomic variation can be a source way which can drives tumor evolution and may provide some potential prognosis information. And a few of studies had addressed the prognostic value of CNA patterns in cancers. As the Fig. 4A shown that the high score group had high significant differential in the genomic variation. Also the high score group had instability genetic alteration, especially the copy number amplification of 13q12.2 and loss of 16p.13.3 in the low score group (Fig. 4B–D). And the mutation frequency of oncogenes had significant differential between the different score groups, our study showed that the TTN, LRP2 and MUC16 had higher mutation frequency in the high score group than the low score group (Fig. 4E,F).

### Validation in external cohorts using ssGSEA

**Figure 5:**
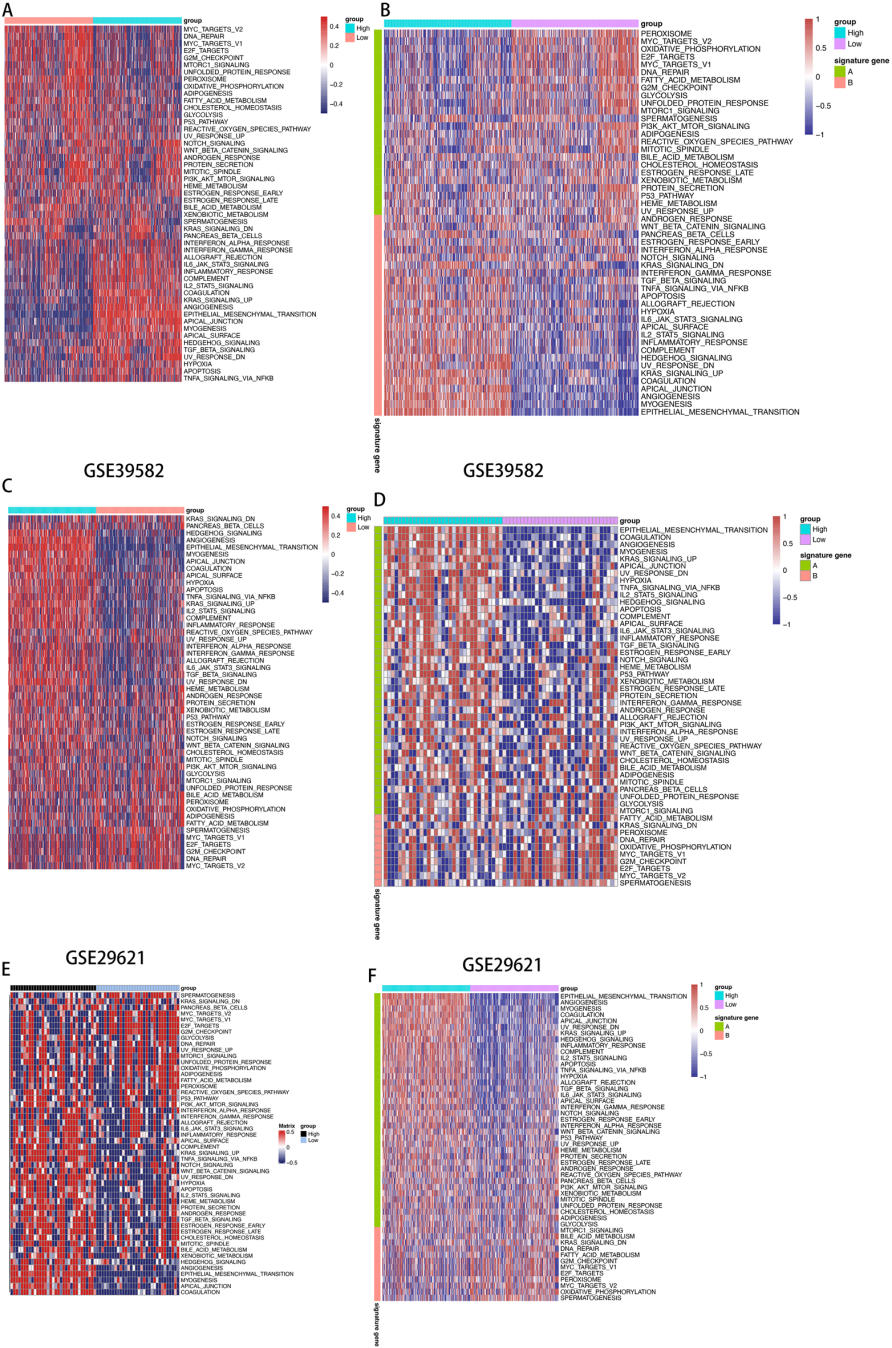
Validation of different score group in external cohorts. (**A,B**) The distribution of various immune cells in the GSE39582 data (**C,D**) and GSE29621 data (**E,F**).

To extend our findings to external cohorts (GSE29621 and GSE39582), we applied the ssGSEA algorithm to the 99 genes in signature A. In both cohorts, the high-score group aligned with the inflammatory subtype, while the low-score group corresponded to the immune desert cluster (Fig. 5C–F). These consistent results further reinforced the robustness of our approach (Fig. 5A,B).

### Uncovering targeted therapies for colon cancer cell lines: insights from drug sensitivity analysis

**Figure 6:**
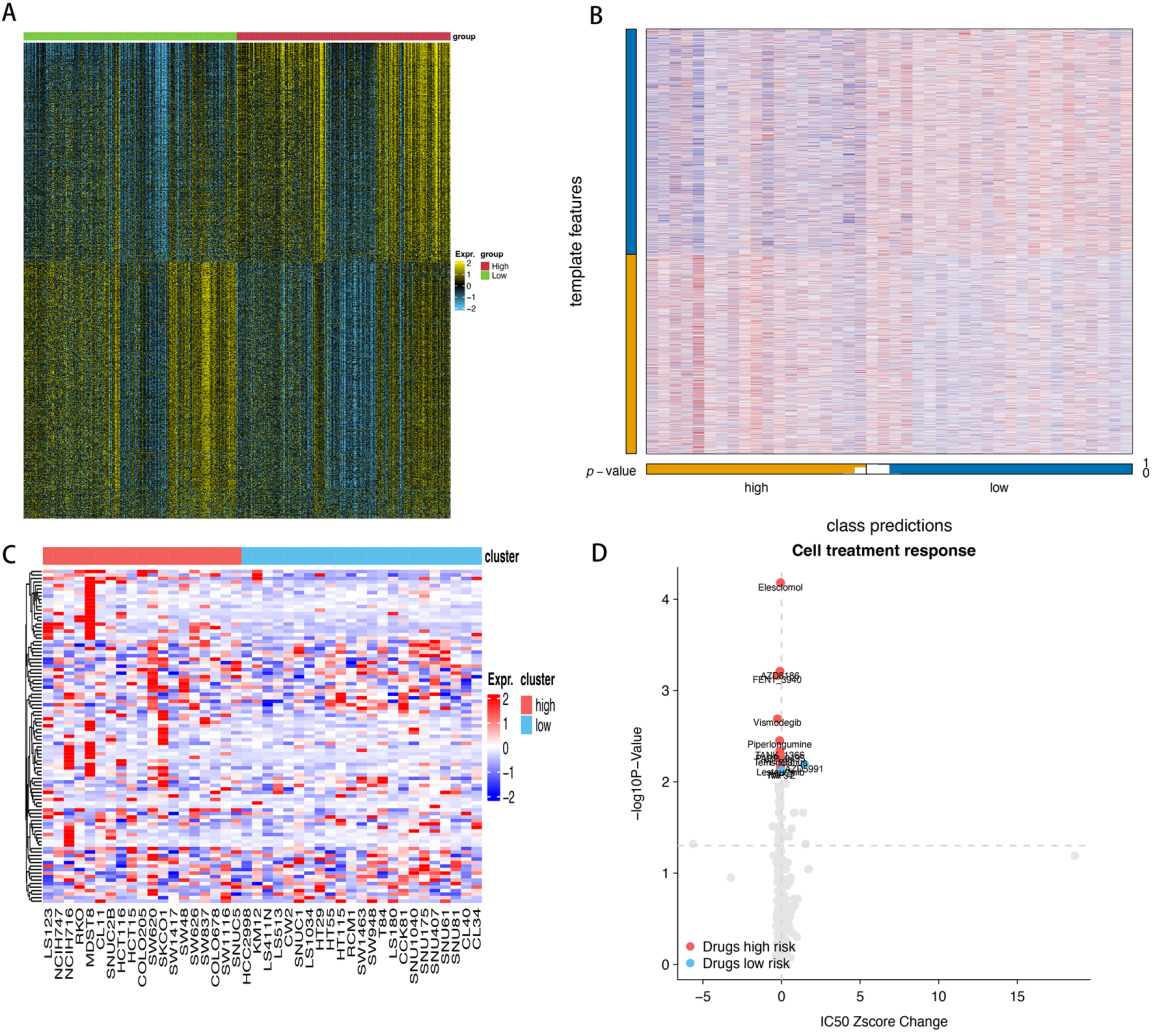
(**A**) Heatmap of subtype-specific upregulated biomarkers using limma for identifying the two clusters. (**B**) Heatmap of NTP. (**C**) Heatmap shows the expression pattern of 99 genes in cancer cell lines showing low or high score group. (**D**) The figure summarizes the relative changes of the ic50z score and P values in selected COAD cell systems at low or high score group which can be treated using the designated compounds based on the cancer drug susceptibility genomics (gdsc) database. The red spot clearly shows the high sensitivity drugs of high score group and the blue point clearly shows the high sensitivity drugs of low score group.

The Genomics of Drug Sensitivity in Cancer (GDSC) dataset was utilized to identify compounds displaying variations in sensitivity among the different score subtypes, aiming to shed light on potentially more effective treatment strategies for both high- and low-score groups. Leveraging 99 signature A genes, we employed the ssGSEA algorithm on colon cancer cell lines to establish a scoring system. Intriguingly, these genes displayed notable expression levels in the transcript profiles across colon cancer cells lines (Fig. 6A). NTP classified each sample in the external cohorts as one of the identified subtypes (Fig. 6B). According to drug sensitivity analysis, the high-score group exhibited significantly increased sensitivity ofElesclomol, TW37, FEN13940 and PI-103 while the low-score group appeared to derive greater benefits from EGFR-target therapy (erlotinib) (Fig. 6C,D) (Table S5).

### Value of scoring in predicting response to immunotherapy

**Figure 7:**
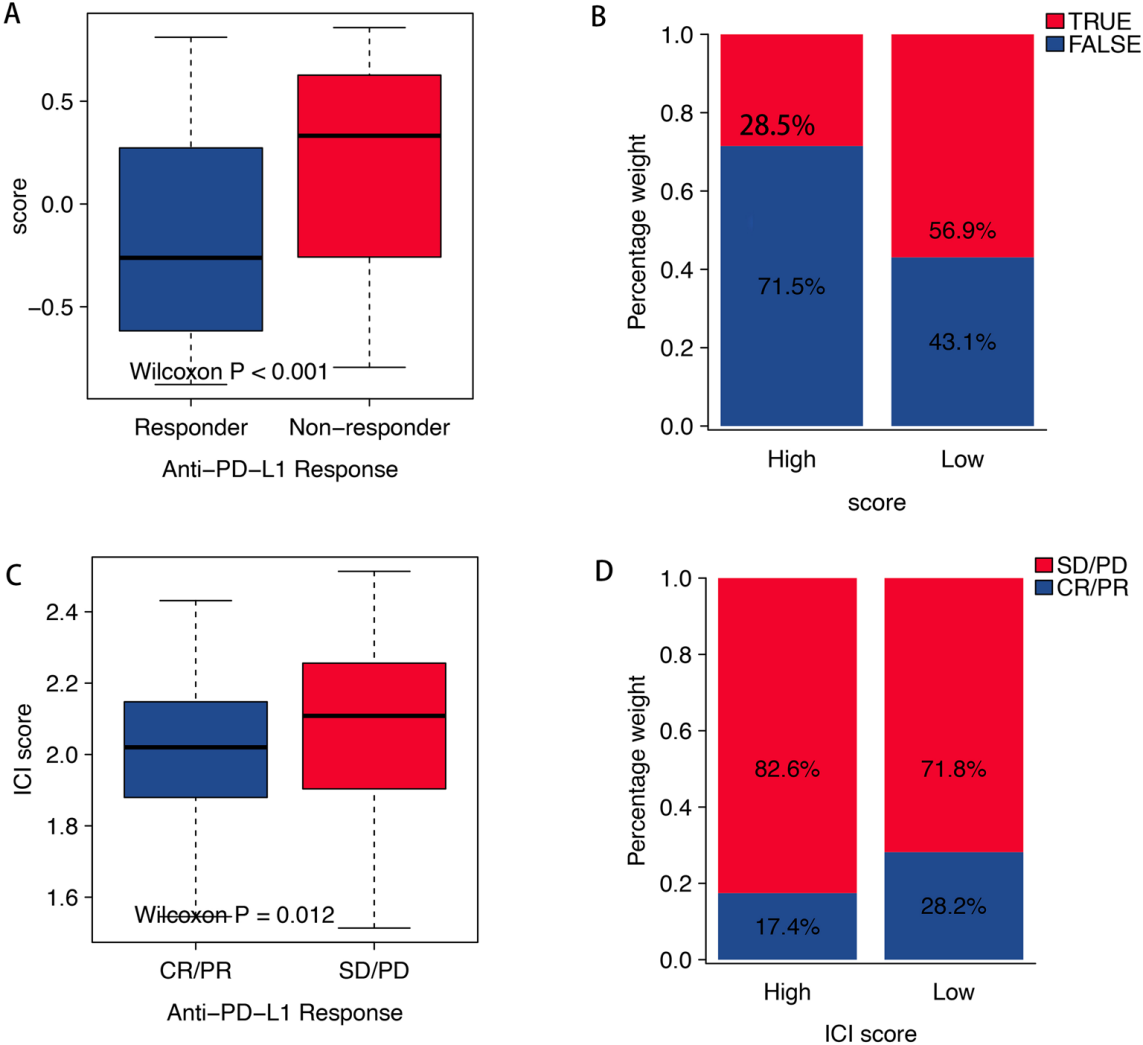
(**A,C**) The distribution of anti-PD-1 response rate in different score groups in the TCGA cohort and IMvigor210 cohort. (**B,D**) The two score groups with different anti-PD-1 response in the TCGA cohort and IMvigor210 cohort.

The application of immunotherapy has greatly improved the survival rate of cancer patients, especially the use of PD-1 or PD-L1 specific monoclonal antibodies. However, the remission rate of immunotherapy remains below 40%. In our study, the value of scoring in immunotherapy in the IMvigor210 cohort and the TCGA cohort was analysed. Here, we preformed the ssGSEA algorithm using 99 signature A genes based on the both cohort to conduct the analyses the effect of anti-PD-L1 immunotherapy in the different score groups. Here, we used the TIDE website to predict the response of cancer immunotherapy with the TCGA database. The rate of objective response in the anti-PD-L1 treatment in the high score group (28.5%) of TCGA was lower than the low score group (56.9%) (Fig. 7B). The IMvigor210 cohort showed the same trend in the objective effectiveness of anti-PD-L1 treatment as the TCGA cohort (high score: 17.4%, low score: 28.2%) (Fig. 7D). At the same time, the score of the response subtype was lower than that of the non-response subtype (Fig. 7A,C).

### The role of BGN gene in colon cancer mechanisms and prognosis

**Figure 8:**
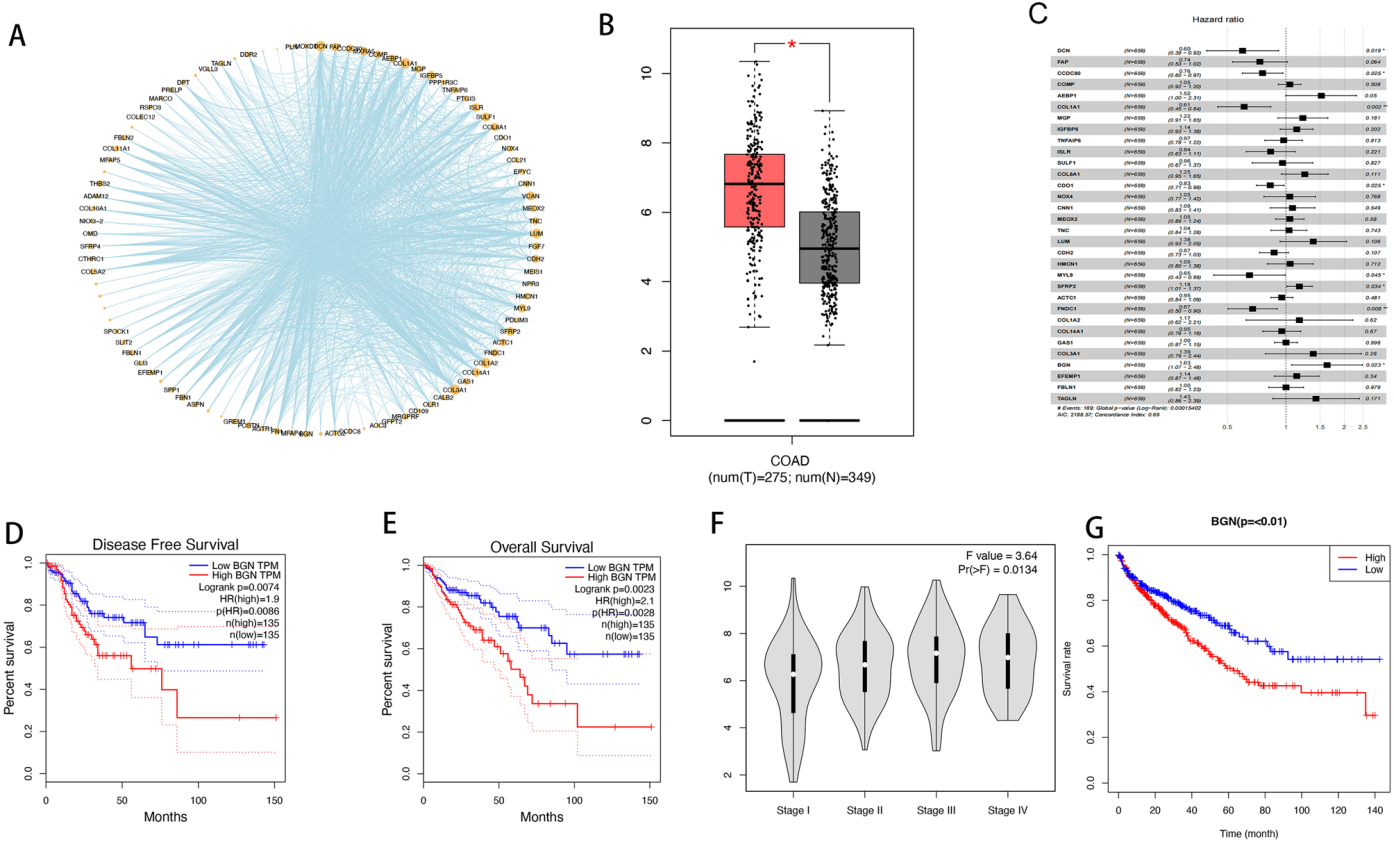
(**A**) PPI network of the signature A genes. (**B**) mRNA expressions of BGN in lung cancer and normal tissues as accessed by GEPIA database. (**C**) Multivariate Cox regression analysis. (**D,E**) The overall survival and diseases free survival analysis of BGN in the GEPIA analysis. (**F**) Box plot of the BGN for different pathological stages in the GEPIA analysis. (**G**) The overall survival analysis of BGN in the COAD.

To delve deeper into the underlying mechanisms, we uploaded the 99 signature A genes into the STRING database to construct a protein–protein interaction (PPI) network, as visualized in Fig. 8A. And the BGN is high expression in the tumor than the normal tissue (Fig. 8B). Our analysis of protein–protein interactions based on STRING database, along with COX analysis, highlighted the pivotal role of BGN genes as key nodes within this network. Univariate Cox regression analysis demonstrated a obust correlation of the BGN gene with OS (HR 1.183, *P* < 0.01) (Fig. 8C, Table S1). Furthermore, Gene Expression Profiling Interactive Analysis (GEPIA) and our research underscored elevated BGN expression is tumor tissues compared to normal ones, with the low expression group exhibiting more favorable outcomes in terms of disease-free survival (DFS) and OS (Fig. 8D,E,G). Multivariate Cox regression analysis identified BGN as an independent prognostic indicator (hazard ratio [HR] 1.63, *P* = 0.02) (Table S2, Fig. 8C). A higher BGN expression level was found in advanced-stage cancer (Fig. 8F). The BGN protein expression levels were examined in 12 colon cancer specimens, we found BGN protein was gradually increase in the normal tissue, stage I, stage II, and stage III tissue, which indicated that the BGN expression in clinical samples may therefore hold prognostic and/or potentially predictive value (Fig. S1).

## Discussion

Colorectal cancer (CRC) stands as the third leading cause of morbidity and mortality as per the 2020 cancer statistics^34^. Colon cancer represents a prevalent subtype of CRC. Recent advancements in therapy and diagnosis have contributed to a substantial reduction in mortality rates. Nevertheless, there remains a notable lack of specificity in colon cancer treatment, leaving ample room for enhancing both therapy and diagnosis. This gap becomes particularly pronounced due to the absence of a clearly defined molecular subtype for this disease. Precision medicine underscores the tailoring of treatments to individual patients, yielding significant benefits that has even been extended to those initially diagnosed with different cancer types. In addition, there is ample evidence indicating that, compared to normal colon tissue, the NF-kB pathway is activated and COX-2 expression is upregulated in stromal myofibroblasts surrounding colonic adenocarcinoma. Given that the upregulation of COX-2 expression is primarily induced by NF-kB, NSAIDs (selective COX-2 inhibitors) may exhibit their chemopreventive properties by directly targeting these cells^35^. Despite the existence of several molecular subtyping approaches, such as CMS, for colon cancer^2^, they primarily target patients with stage I to III colon cancer and have yet to find widespread application in clinical treatment. This indicates a need for further research into potential molecular subtyping within the context of colon cancer, aimed at refining the precision of treatment for patients.

Advances in bioinformatics, particularly high-throughput sequencing technology, allow for the investigation into molecular mechanisms underlying malignancies at the transcriptome level. Researchers have harnessed these tools to develop signatures for improved prognosis estimation and to uncover the underlying mechanisms of diverse cancers^36–42^. Moreover, clustering analysis categorizes cancer patients into molecular subtypes based on specific gene sets, known as reference points. The selection of these reference gene sets is crucial for determining the quality of molecular subtyping.

A large number of studies on molecular typing of COAD based on omics or specific gene sets have emerged. Recently, Yang integrated the multi-omics data of all COAD patients based on a single algorithm, but the analysis did not include immune cell infiltration data^43^. Chen et al. integrated the multi-omics data of COAD for reclassification, but this study only classified from each omics level, without realizing the real integration of multi-omics data for classification^44^. Although these studies provide new directions for the diagnosis and treatment of COAD to some extent, there are also some shortcomings. The classification methods used in most typing studies are relatively simple. These shortcomings make it difficult to apply these classification studies to clinical practice. The development of multi-omics makes it easier for researchers to deepen their understanding of cancer at the molecular level. At the same time, a large amount of omics data also brings new challenges to analysts^45^. It is particularly crucial to reduce data noise and obtain the key features of tumor occurrence and development while retaining tumor characteristics^45^. At present, there are few studies trying to establish a comprehensive model based on multi-omics data to predict the prognosis and personalized drug selection of COAD patients. Therefore, it is particularly important to develop a comprehensive and reliable model for prognosis and drug selection of COAD patients to assist prognosis prediction and guide personalized treatment. Previous studies mainly focused on the expression of certain specific genes to predict the prognosis of tumors^46–48^, however, the biological process of tumors is extremely complex, and different types of features are interrelated. Using multi-omics analysis to explain the heterogeneity of tumors and constructing relevant models to predict the efficacy of immunotherapy can make the system more stable and convincing. This process not only reduces the untruthfulness of the results of CIBERSORT^49^, but also makes the model system stable, free from the influence of single or multiple gene expressions^50–52^. Our study, a set of 200 EMT-related genes and data on 22 immune cell types were utilized to establish a connection between EMT and immune cell populations. Leveraging these two datasets, we applied the MOVICS algorithm to delineate distinct molecular subtypes. Our findings revealed that cluster B exhibited heightened activity within EMT pathways which, unfortunately, correlated with a poorer prognosis. Building upon this, we sought to understand the relationship between DEGs, 22 immune cells, tumorigenesis-related pathways, and the identified clusters by conducting Pearson correlation analysis. Through this analysis, we identified 99 genes, M2 macrophages, and M1 macrophages as the markers of cluster B (Tables S3, S4). The ssGSEA applied for quantifying clusters a strong overlap between the high-score group and cluster B. Intriguingly, a poorer survival outcome was observed in the high-score group which displayed heightened activation of pathways related to tumorigenesis, including EMT, angiogenesis, coagulation, and myogenesis. To ensure the robustness of our approach, we validated our scoring system in two external cohorts (GSE29621 and GSE39582) to predict the activity of tumorigenesis pathways of colon cancer patients. Encouragingly, our results demonstrated the accuracy and reliability of our scoring method. Moreover, driver gene distribution in both high- and low-score groups was investigated using MAFtools. Among the top 25 genes with the highest frequency, we observed significant differences between the two groups. These findings not only contribute valuable insights into the mechanisms governing tumor components but also shed light on gene mutations relevant to immune therapy.

GDSC data were harnessed to identify potential compounds effective for distinct score groups. Our findings unveiled that AZD5991, functioning as an MCL inhibitor, exhibited heightened sensitivity in the low-score group, while the high score group displayed increased sensitivity to PI-103, a dual PI3K and mTOR inhibitor.

In summary, our research leveraged multi-omics data to construct a predictive model for colon cancer prognosis, successfully identifying the high-score group as characterized by elevated EMT activity. Our study established a scoring model capable of forecasting EMT activity and prognosis, revealing a greater benefit from MCL inhibition within the high-score group. Furthermore, our investigation unveiled diverse cellular and molecular stratifications among colon cancer patients, suggesting that the high-score group derive substantial advantages from dual inhibition of PI3K and mTOR by PI-103. Our study presents evidence indicative of specific epigenomic alterations preceding distinct prognosis groupings, thereby enhancing the early classification of cancer patients. Furthermore, our research revealed the elevated expression of BGN as a distinctive feature associated with the high activity of EMT phenotype and gradually increase in the normal tissue, stage I, stage II, and stage III tissue, underscoring its potential as an attractive medication target in combination therapy approaches.

Our model is effective in predicting prognostic efficacy, but the scoring model needs to be confirmed by prospective analysis of a large cohort of patients with COAD, and the efficacy of the model needs to be validated in appropriate preclinical models and future clinical trials.

### Supplementary Information

Supplementary Figure S1.

Supplementary Tables.

## Data Availability

Publicly available datasets were analyzed in this study. This data can be found here: The Cancer Genome Atlas (TCGA) database (https://portal.gdc.cancer.gov/) and Gene Expression Omnibus (https://www.ncbi.nlm.nih.gov/geo/).
