# Supplementary material for: Exploring the mystery of colon cancer from the perspective of molecular subtypes and treatment
Source: Sci Rep. 2024 May 13;14:10883. doi: 10.1038/s41598-024-60495-8 (PMC11091141; doi:10.1038/s41598-024-60495-8)
Supplement: Supplementary file 1 — Supplementary Figure S1. [file 41598_2024_60495_MOESM1_ESM.docx]

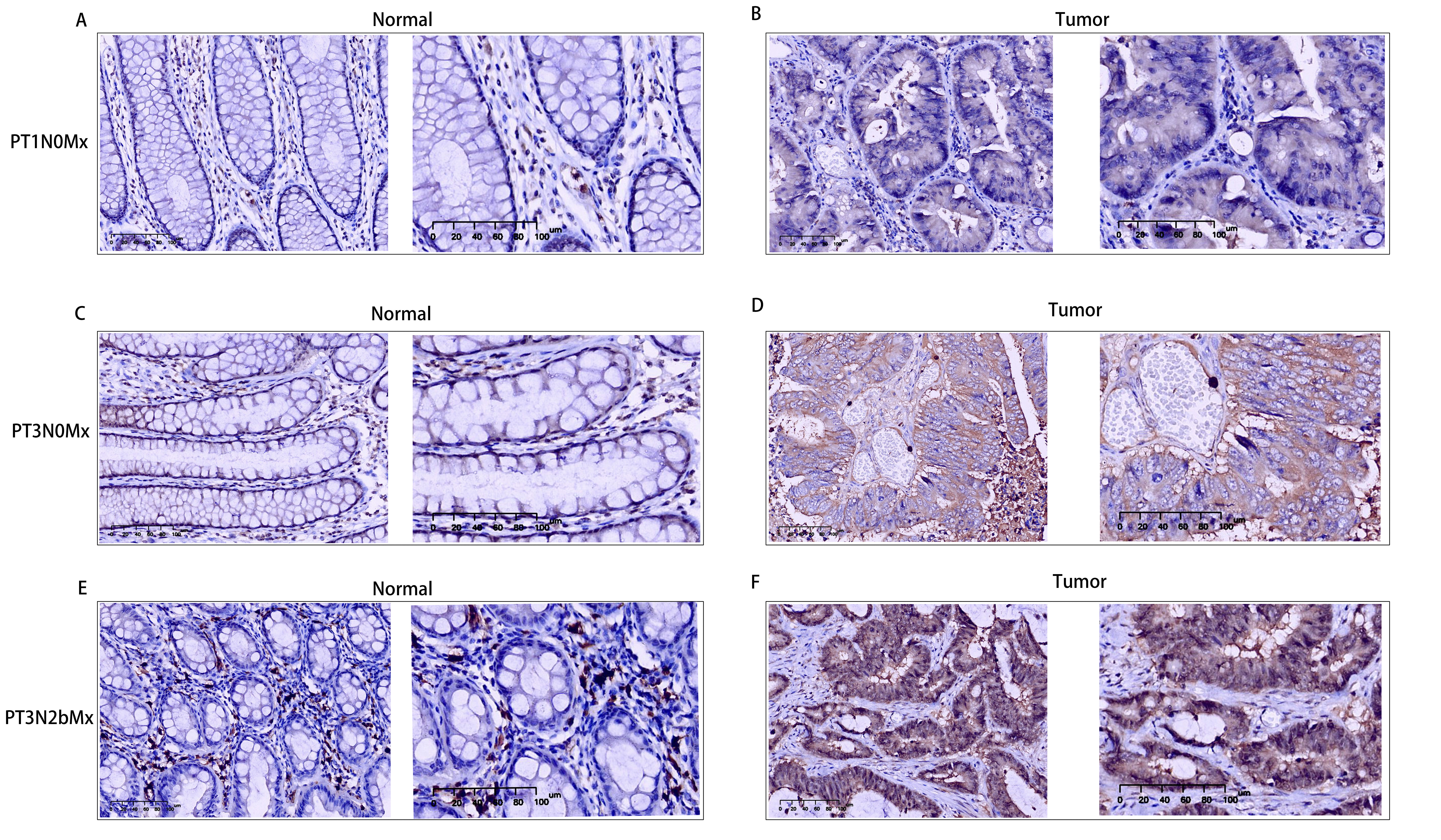


**Figure S1** A-B: The BGN protein expression levels of stage I is higher than normal tissue(adjacent tissues); C-D: The BGN protein expression levels of stage II is higher than normal tissue(adjacent tissues); E-F: The BGN protein expression levels of stage III is higher than normal tissue(adjacent tissues).
